# Evidence for a genetic sex determination in Cnidaria, the Mediterranean red coral (*Corallium rubrum*)

**DOI:** 10.1098/rsos.160880

**Published:** 2017-03-01

**Authors:** M. Pratlong, A. Haguenauer, S. Chenesseau, K. Brener, G. Mitta, E. Toulza, M. Bonabaud, S. Rialle, D. Aurelle, P. Pontarotti

**Affiliations:** 1 Aix Marseille Univ, Avignon Université, CNRS, IRD, IMBE, Marseille, France; 2 Aix Marseille Univ, CNRS, Centrale Marseille, I2M, Marseille, France; 3 Perpignan Via Domitia Univ, IHPE UMR 5244, CNRS, IFREMER, Montpellier Université, Perpignan, France; 4 UMS BioCampus- MGX Montpellier GenomiX, Institut de Génomique Fonctionelle, 141 rue de la Cardonnille, 34094 Montpellier Cedex 05, France

**Keywords:** genetic sex determination, *Corallium rubrum*, RAD-sequencing, Cnidaria

## Abstract

Sexual reproduction is widespread among eukaryotes, and the sex-determining processes vary greatly among species. While genetic sex determination (GSD) has been intensively described in bilaterian species, no example has yet been recorded among non-bilaterians. However, the quasi-ubiquitous repartition of GSD among multicellular species suggests that similar evolutionary forces can promote this system, and that these forces could occur also in non-bilaterians. Studying sex determination across the range of Metazoan diversity is indeed important to understand better the evolution of this mechanism and its lability. We tested the existence of sex-linked genes in the gonochoric red coral (*Corallium rubrum*, Cnidaria) using restriction site-associated DNA sequencing. We analysed 27 461 single nucleotide polymorphisms (SNPs) in 354 individuals from 12 populations including 53 that were morphologically sexed. We found a strong association between the allele frequencies of 472 SNPs and the sex of individuals, suggesting an XX/XY sex-determination system. This result was confirmed by the identification of 435 male-specific loci. An independent test confirmed that the amplification of these loci enabled us to identify males with absolute certainty. This is the first demonstration of a GSD system among non-bilaterian species and a new example of its convergence in multicellular eukaryotes.

## 1. Introduction

Sexual reproduction is ubiquitous among eukaryotes [1], and there is a wealth of literature on the evolutionary advantages of sex [2]. Although sex is widely shared, the corresponding mechanisms and the sex-determination systems vary greatly among species [3,4]. In gonochoric species, the sexual identity of individuals is defined by sex-determination systems, going from purely genetic sex determination (GSD), to purely environmental sex determination (ESD) where the same genotype can produce both male and female phenotypes depending on environmental conditions [3]. GSD is observed, for example, in mammals, where sex chromosomes are present. The echiurian *Bonellia viridis* gives an example of ESD, where larvae recruiting on a female will develop into males, but otherwise become female [5]. While GSD has been intensively studied in bilaterian species, one example has been recorded so far among non-bilaterians such as Porifera, Cnidaria and Ctenophora [3,4]. However, the quasi-ubiquitous repartition of GSD among eukaryotes suggests that similar evolutionary forces repeatedly led to the evolution of GSD, and these forces could occur also in non-bilaterians. This lack of evidence of GSD in non-bilaterians is probably the consequence of a reduced number of model organisms in these groups, and sometimes of the difficulty to identify separate sexes. Nevertheless, studying sex determination along the range of Metazoan diversity is important to understand better the evolution of this mechanism and its lability. For example, understanding the ancestral state of sex-determination systems in Metazoans requires studying them in the main branches of the phylogenetic tree.

Cnidarians display various sexual systems, from hermaphrodism (simultaneous or sequential) to gonochorism, and sexual reproduction can take place at different stages, polyp or medusae [6]. The corresponding system determinations are poorly known: a few ESD examples have been reported but no examples of GSD have so far been confirmed among non-bilaterians [6]. A cytogenetic analysis has shown a clear evidence of potential sex chromosomes in a scleractinian [7] but the role of these chromosomes in sex determination remains to be studied as this species is hermaphroditic. Gonochorism is highly predominant in octocorals (89% of the species [8]), even if cases of rapid transition between gonochorism and hermaphrodism have been demonstrated in the genus *Alcyonium* [9]. The relative stability of gonochorism in octocorals makes them interesting models for the study of sex-determination systems. This could, for example, correspond to an evolutionary trap, which would stabilize a sex-determination system [4], or, conversely, to a variety of sex-determination systems, but with a selective pressure for gonochorism.

The red coral (*Corallium rubrum*) is a long-lived gonochoric octocoral, with an age at first reproduction of around 7–10 years [10,11]. This harvested species is the object of ecological and population genetic studies [12,13] for conservation and management purposes. Although hermaphrodite colonies have been mentioned in the first description of the reproduction of this species [14], it is considered to be gonochoric. Red coral individuals from the two sexes are morphologically identical at the macroscopic scale, and the sex can be identified microscopically after dissection only during the period of gametogenesis (from May to September) [15]. Elucidating the sex-determination factors in this species would be useful for a better understanding of its biology and of its potential response to environmental change. It would also widen our knowledge of sex-determination systems in cnidarians. The identification of GSD may be difficult in groups where cytogenetic analyses are problematic to implement, as is often the case in non-model species, or when species lack visually heteromorphic sex chromosomes [16,17]. Thus, our goal here was to test the existence of sex-linked genes in the precious red coral, with a population genomic approach. We used restriction site-associated DNA sequencing (RAD-Seq) applied to sexed individuals, as proposed in [6]. Our results point to an XX/XY sex-determination system in this species, and we developed a polymerase chain reaction (PCR)-based protocol for sexing.

## 2. Material and methods

### 2.1. Sampling and DNA extraction

*Corallium rubrum* colonies were collected by scuba diving at two depths of two sites in three geographical regions of the Mediterranean Sea (Marseille, Banyuls, Corsica) between February and August 2013 (electronic supplementary material, figure S1 and table S1). Thirty individuals per site and depth were collected (360 individuals), preserved in 95% ethanol and stored at −20 °C. To validate our results, 40 additional individuals were collected in one site near Marseille in June 2016 and conserved in both 95% ethanol and formaldehyde. Total genomic DNA was extracted according to the protocol of [18], followed by a purification using Qiagen DNeasy blood and tissue spin columns.

### 2.2. Morphological sex identification

Samples fixed in 95% ethanol from the Corsica populations were decalcified in 10% EDTA adjusted to a pH of 7.4 with NaOH for 48 h and dehydrated in graded alcohols, cleared in xylene substitute (Neo-clear VWR) and embedded in paraffin wax. Sections (7 µm) were cut using a rotary microtome. Sections were stained using trichrome of Masson variant of Goldner protocols and examined using a Leica DMLB. The sex of individuals from the Marseille population collected in June 2016 and preserved in formaldehyde was identified under a dissecting microscope after dissection.

### 2.3. Restriction site-associated DNA sequencing

Twelve RAD libraries were prepared according to the protocol described in [19], with small modifications (see the electronic supplementary material, methods). Libraries were sequenced on an Illumina HiSeq2000 using 100 bp single-end reads, at the Biology Institute of Lille (IBL, UMR 8199 CNRS) and at the MGX sequencing platform in Montpellier (France). The Stacks pipeline [20,21] was used for the loci de novo assembly and genotyping. We applied several filters to the resulting dataset in order to filter for poor-quality single nucleotide polymorphisms (SNPs) and artefacts due paralogous sequences (electronic supplementary material, table S2 and methods).

### 2.4. Identification of sex-linked loci

**Figure 1.**
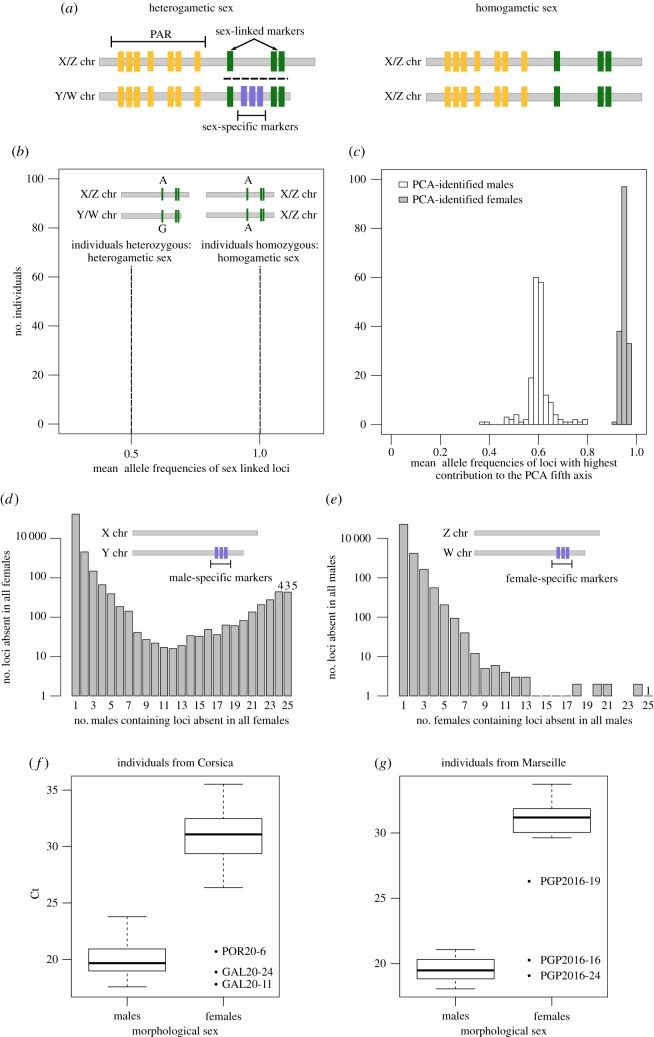
(*Caption* *opposite*.)

The presence of sex-linked loci, i.e. loci common to the two sex chromosomes but presenting differences in allele frequencies between sex (figure 1*a*), was explored by performing a principal component analysis (PCA) using the package adegenet in R [22,23]. This analysis was performed on the total dataset (12 populations). The dataset was centred and missing data were replaced by the mean allele frequency for each locus.

**Figure 1.** (*Opposite*.) (*a*) Example of a genetic sex-determination system. Sex-specific markers (blue) are present on the Y chromosomes in the XX/XY system and in the W chromosome in the ZW/ZZ system. Sex-linked markers (green) are common to the two sex chromosomes but should present differences in allele frequencies between sex because of the recombination arrest (adapted from [15]). (*b*) Mean allele frequencies expected in the case of sex-linked markers fixed on the X/Z and on the Y/W chromosomes. For those markers, individuals of the heterogametic sex (males in the XX/XY system, females in the ZW/ZZ system) should be heterozygous and individuals of the homogametic sex (females in the XX/XY system, males in the ZW/ZZ system) should be homozygous. (*c*) Mean allele frequencies of loci with the highest contribution to the PCA fifth axis observed in PCA-identified males and females. (*d*,*e*) Distribution of loci absent in all morphologically sexed individuals of one sex in morphologically sexed individuals of the opposite sex. (*f*) Results of the real-time PCR amplification (threshold cycle Ct) of one male-specific locus (Locus_139082) on morphologically sexed males and females from the Corsica populations. The Ct indicates the number of PCR cycles necessary to reach a threshold value; a low Ct value indicates a high amplification rate from the corresponding sample. Results for the six loci tested were similar (electronic supplementary material, figure S3). (*g*) Result of the real-time PCR amplification (threshold cycle Ct) of one male-specific locus (Locus_139082) on morphologically sexed males and females from the Marseille population. Results for the six loci tested were similar (electronic supplementary material, figure S4).

### 2.5. Identification of sex-specific loci

We filtered the Stacks catalogue in order to search among morphologically sexed individuals for loci present in all individuals from one sex (one read or more by individual) and absent in all individuals from the other sex (no read detected) (i.e. sex-specific loci; figure 1*a*).

### 2.6. Real-time polymerase chain reaction

Primers were designed for six putative male-specific sequences using IDT online tool (http://eu.idtdna.com/Primerquest/Home/Index) (primer sequences in electronic supplementary material, table S3). To avoid a specific hybridization, we selected loci presenting no blast hit results other than themselves against the Stacks catalogue. The presence or absence of target genomic sequences was assessed by real-time PCR on 1 µl of the same DNA extracts of the 58 morphologically sexed individuals from Corsica used for RAD-Seq, and for the 39 supplementary morphologically sexed individuals from Marseille (see the electronic supplementary material, methods). The difference of amplification between males and females was tested with a Wilcoxon–Mann–Whitney test in R [23].

## 3. Results and discussion

### 3.1. Morphological identification of sex

The sexing of the 58 individuals from four populations in Corsica was undertaken on the basis of gonad analysis. Among them, 25 males and 28 females were formally identified (hereafter called morphological males and females). Five individuals were sexually undetermined because of the quality of the tissues, or due to a sexually immature stage of development. Among the 40 individuals from the sampling of June 2016 in Marseille, we identified 15 males and 24 females (one individual was sexually undetermined).

### 3.2. RAD-tag sequencing and quality filtering

RAD-tag sequencing generated an average of 187 ± 21 million reads per library before any quality filtering. The quality filtering step enabled us to remove an average of 2.02% of reads without a correct restriction enzyme cut site, an average of 1.69% of reads with ambiguous barcodes and an average of 1.61% of reads with low quality score. An average of 183 ± 22 million reads per library (95% of total reads) were retained (electronic supplementary material, table S4) with an average of 5.7 million reads per individual. After assembly (see details in electronic supplementary material, methods), 138 810 SNPs were successfully genotyped in at least 75% of individuals from all populations (electronic supplementary material, table S2). Individuals were sequenced with a mean coverage of 37 reads per individual per locus. Morphologically sexed females presented a mean coverage of 37.9 reads per individual per locus, and males 45.6 reads per individual per locus. A total of 27 461 SNPs remained after several steps of filtration (electronic supplementary material, table S2 and methods).

### 3.3. Identification of sex-linked loci

**Figure 2.**
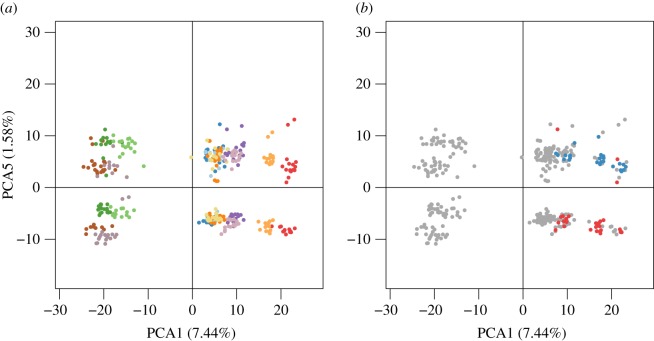
Principal component analysis (axes 1 and 5) of the 12 red coral populations (*n* = 354 individuals, 27 461 SNPs). Colours correspond to the (*a*) population of individuals (light blue: BANN20, dark blue: BANN40, dark orange: BANS20, yellow: BANS40, brown: ELV12, grey: MEJ40, dark green: FIG8, light green: MOR40, red: GAL20, light orange: GAL40, dark purple: POR20, light purple: POR40; see electronic supplementary material, figure S1 and table S1) and (*b*) sex of individuals determined morphologically (red: females, blue: males).

While the first four principal components of the PCA highlighted neutral population genetic differences [24], the fifth axis (1.58% of explained variance) separated individuals from all populations in two clear groups that matched the male/female repartition of morphologically sexed individuals (figure 2). Only three individuals that were morphologically female appeared among males in the PCA. From this PCA, we identified potential females and males among individuals for which sex determination was not performed on the basis of the visible separation in two groups. Considering this separation, we postulate that the dataset comprised 169 females and 185 males, corresponding to a balanced sex ratio (*p* = 0.40, electronic supplementary material, table S5). The sex ratio was balanced also inside each population, except in ELV12, where it was significantly biased towards male individuals (70% of males; *p* = 0.03, electronic supplementary material, table S5). There were 472 SNPs with a contribution to the fifth axis of the PCA higher than 1% (electronic supplementary material, figure S2); these loci displayed the same allele fixed in almost all morphological females, and were at the heterozygous state in almost all morphological males. This observation was confirmed with PCA-identified males and females (figure 1*b*,*c*). The genotypes of these 472 SNPs enabled the sexing of 95% of individuals for the whole dataset. Among these SNPs, when considering only morphologically sexed individuals, 379 SNPs were fixed in all females, 59 were at the heterozygous state in all males (the remaining SNPs were at the heterozygous state in almost all males). Fifty-five SNPs were in these two categories and were therefore diagnostic of sex for the morphologically sexed individuals. Identification of such SNPs that were homozygous in females and heterozygous in males suggested an XX/XY sex-determination system in the red coral, with a non-recombining XY-like region. Of the SNPs leading the fifth PCA axis, 347 were fixed in all PCA-identified females. However, none of these SNPs was at the heterozygous state in 100% of PCA-identified males. SNPs that were strictly homozygous in females and heterozygous in males were found inside each population and geographical region, but none was common to the three geographical regions. Furthermore, even if we did not observe markers that were heterozygous in 100% of males, these markers remain at the heterozygous state in the majority of males: 303 of these 472 sex-linked markers were at the heterozygous state in more than 70% of all males. The absence of markers diagnostic of sex in the overall dataset may indicate that these loci were submitted to a low but non-null recombination rate, as is the case when sexual chromosomes have recently diverged, or near the boundary of pseudoautosomal regions, where recombination is more frequent than in fully sex-linked regions [25,26]. It could also be the result of polymorphism within the restriction sites on the Y chromosome (i.e. allele dropout) making it impossible to observe the Y allele with RAD-Seq in some populations [27]. A low rate of mutation, recombination or allele dropout could suffice to make a marker go from diagnostic (heterozygous in 100% of males) to sex-linked but not diagnostic (heterozygous in less than 100% of males). For these different reasons, it may be difficult to identify strictly diagnostic loci common to all populations for such species with a strong genetic structure. Nevertheless, even if diagnostic markers of sex were not found at the scale of the overall dataset, the multilocus analysis enabled us to identify clearly males and females. Sex-linked polymorphisms identified by RAD-Seq have been found in the pistachio (*Pistacia vera*) and the salmon louse (*Lepeophtheirus salmonis*), where authors identified markers being heterozygous in females and homozygous in males, and suggested a ZW/ZZ system [28,29]. These polymorphisms have also been found in the Atlantic halibut (*Hippoglossus hippoglossus*), the Nile tilapia (*Oreochromis niloticus*) and the date palm (*Phoenix dactylifera*), where an XX/XY system was suggested [30–32]. Here, we highlighted a strong signal of sex-linked markers in our population genomic dataset. Considering the pattern of allele frequencies of these loci, they may be detected as being under balanced selection, which can easily lead to a misinterpretation of the signal when these loci are not expected (especially in species whose sex-determining system is unknown).

### 3.4. Identification of sex-specific loci

We identified 435 loci present in all 25 morphological males and absent in the 28 morphological females (figure 1*a*,*d*,*e*). To avoid any bias caused by an eventual misidentification of the sex of the three misclassified females, these individuals have not been taken into account for this analysis. To confirm the male specificity of these 435 loci, we targeted six of them in real-time PCR of morphologically sexed individuals. The six loci could be amplified in 100% of morphologically sexed males. Almost no amplification signal was observed in 100% of morphologically sexed females (figure 1*f*; electronic supplementary material, figure S3; *p* < 0.01 in all tests, electronic supplementary material, table S6). The presence of male-specific loci supports our previous hypothesis of a system with male heterogamety (i.e. XX/XY) [16,17,33]. Finally, in order to confirm that the male-specific markers identified from only one geographical region were not the result of random divergence between sexes, we applied the real-time PCR test to the 40 individuals from the additional June 2016 sampling in Marseille. The six male-specific primer pairs enabled us to amplify 100% of morphologically sexed males by real-time PCR. Almost no signal was observed in 22 of the morphologically sexed females (figure 1*g*; electronic supplementary material, figure S4; *p* < 0.01 in all tests, electronic supplementary material, table S6). Two females presented an amplification profile of the six markers similar to that of the males, as was also the case for three females from Corsica. This confirms that these loci are male-specific, and that they are conserved between the two distant populations.

### 3.5. Cross-validation

**Figure 3.**
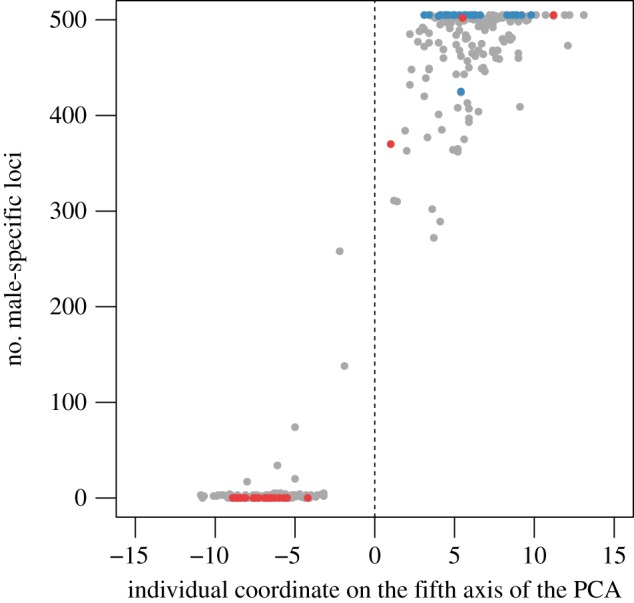
Plot of the number of male-specific loci possessed by an individual as a function of the individual coordinate on the fifth axis of the PCA. Morphologically sexed individuals are indicated (red: females, blue: males).

Finally, as a validation test, we crossed the results obtained by PCA and by sex-specific loci by searching the presence of the 435 male-specific loci in all 354 individuals (figure 3). Twenty-four per cent of PCA-identified males possessed all 435 male-specific loci and 80% of the individuals possessed 90% of these loci. Some of these loci may have been lost during library preparation and sequencing, or due to allele dropout, thereby explaining their absence in some of the males. Furthermore, the male-specific loci have been found from 25 individuals, and it is likely that some of them have been detected erroneously if the pattern of technical missing data for a locus followed by chance the male/female distribution. All of the 435 male-specific loci were absent in 40% of PCA-identified females, and 97% of PCA-identified females contained less than 1% of male-specific loci. Figure 3 illustrates the correlation between the three methods of sex identification presented here (coordinate on the fifth axis of the PCA, number of male-specific loci and morphological identification). We observed an extremely good correlation between these three methods, the groups of males and females being well defined in each case. The three misclassified females identified from the PCA also possessed a high number of male-specific loci, and seemed to be genetically male. Such incongruence between sex genotype and phenotype may result from an environmental sexual reversal during sexual differentiation [34], or from the existence of females with the XY genotype, as already described in several mammals [35]. Finally, four individuals that have not been sexed morphologically presented female genotypes but between 15 and 65 male-specific loci. Further analysis is needed to determine the sexual identity of these individuals.

The number of sex-specific markers should depend on the size of the non-recombining region and on the divergence between X and Y, from a minimal Y-specific region for homomorphic sex chromosomes to several sex-specific markers in heteromorphic sex chromosomes [33]. The 435 male-specific loci detected here suggested at least 218 male-specific PstI sites, which is far higher than the number of sex-specific loci in other similar studies based on RAD-Seq [17,33,36]. However, considering the specificity of each study (genome size, frequency of the restriction site and parameters used for the loci assembly), we could not directly compare the number of sex-linked markers or the number of male-specific markers.

Finally, the functional annotation of loci in the Stacks catalogue (see the electronic supplementary material, methods) enabled us to identified a homologue of a double-sex and mab3-related transcription factor (*Dmrt*). The *Dmrt* family of transcription factors is involved in sex determination in numerous metazoans, and the conservation of this function in cnidarians has recently been shown [37,38]. In our case, this gene was neither a sex-specific marker nor a sex-linked marker, which remains compatible with a role in sexual differentiation.

## 4. Conclusion

In summary, the exploration both of sex-linked polymorphisms and of sex-specific loci enabled us to identify an XX/XY genetic sex-determination system in the red coral. This is the first time such an identification has been made for non-bilaterian species, and is a new example of the evolution of GSD in multicellular organisms [3,39]. As gonochorism is predominant among octocorals [8], they constitute a promising group for further exploration of GSD among Cnidaria. In hexacorals, the sister-group of octocorals [40,41], there is apparently a higher diversity of reproduction systems than in octocorals [6]. Extending our study to other anthozoans would be useful in order to test scenarios of evolution of reproduction systems in this group [6]. Furthermore, our study has shown that signal of GSD, if it is unexpected, can be misinterpreted in non-model organisms (for example because sex-linked markers could be detected as being under balanced selection). Our research also emphasizes that population genomic datasets should be analysed and interpreted by taking into account the possibility of GSD in non-model organisms. We provide here real-time PCR primers that will enable the identification of red coral males and females, and that will facilitate the monitoring of the population dynamics of this emblematic species, which is being increasingly submitted to anthropic pressures such as harvesting and global change [42–44].

## Supplementary Material

Supplementary figures

## Supplementary Material

Supplementary tables

## Supplementary Material

Supplementary methods (libraries preparation and analysis of RAD-Sequencing and real-time PCR protocol)

## Data accessibility

The raw DNA sequences are available in the Short Read Archive (SRA) database under accession no. SRR5186771-SRR5187129 [45]. The filtered SNP file is available from the Dryad Digital Repository: http://dx.doi.org/10.5061/dryad.rs7bm [46].

## References

[1] Speijer D, Lukeš J, Eliáš M 2015 Sex is a ubiquitous, ancient, and inherent attribute of eukaryotic life. Proc. Natl Acad. Sci. USA 112, 8827–8834. (doi:10.1073/pnas.1501725112) 26195746 10.1073/pnas.1501725112 PMC4517231

[2] Otto SP 2009 The evolutionary enigma of sex. Am. Nat. 174, S1–S14. (doi:10.1086/599084) 19441962 10.1086/599084

[3] Beukeboom LW, Perrin N 2014 The evolution of sex determination. Oxford, UK: Oxford University Press.

[4] Bachtrog D et al. 2014 Sex determination: why so many ways of doing it? PLoS Biol. 12, e1001899 (doi:10.1371/journal.pbio.1001899) 24983465 10.1371/journal.pbio.1001899 PMC4077654

[5] Berec L, Schembri PJ, Boukal DS 2005 Sex determination in *Bonellia viridis* (Echiura: Bonelliidae): population dynamics and evolution. Oikos 108, 473–484. (doi:10.1111/j.0030-1299.2005.13350.x)

[6] Siebert S, Juliano CE In press Sex, polyps, and medusae: determination and maintenance of sex in cnidarians. Mol. Reprod. Dev. (doi:10.1002/mrd.22690) 10.1002/mrd.22690 27531602

[7] Taguchi T et al. 2014 Molecular cytogenetic analysis of the scleractinian coral *Acropora solitaryensis* Veron & Wallace 1984. Zool. Sci. 31, 89–94. (doi:10.2108/zsj.31.89) 24521318 10.2108/zsj.31.89

[8] Kahng SE, Benayahu Y, Lasker HR 2011 Sexual reproduction in octocorals. Mar. Ecol. Prog. Ser. 443, 265–283. (doi:10.3354/meps09414)

[9] McFadden CS, Donahue R, Hadland BK, Weston R 2001 A molecular phylogenetic analysis of reproductive trait evolution in the soft coral genus *Alcyonium*. Evolution 55, 54–67. (doi:10.1111/j.0014-3820.2001.tb01272.x) 11263746 10.1111/j.0014-3820.2001.tb01272.x

[10] Torrents O, Garrabou J, Marschal C, Harmelin J 2005 Age and size at first reproduction in the commercially exploited red coral *Corallium rubrum* (L.) in the Marseilles area (France, NW Mediterranean). Biol. Conserv. 121, 391–397. (doi:10.1016/j.biocon.2004.05.010)

[11] Marschal C, Garrabou J, Harmelin J, Pichon M 2004 A new method for measuring growth and age in the precious red coral *Corallium rubrum* (L.). Coral Reefs 23, 423–432. (doi:10.1007/s00338-004-0398-6)

[12] Ledoux J-B, Mokhtar-Jamaï K, Roby C, Féral J-P, Garrabou J, Aurelle D 2010 Genetic survey of shallow populations of the Mediterranean red coral [*Corallium rubrum* (Linnaeus, 1758)]: new insights into evolutionary processes shaping nuclear diversity and implications for conservation. Mol. Ecol. 19, 675–690. (doi:10.1111/j.1365-294X.2009.04516.x) 20074314 10.1111/j.1365-294X.2009.04516.x

[13] Santangelo G, Bramanti L, Iannelli M 2007 Population dynamics and conservation biology of the over-exploited Mediterranean red coral. J. Theor. Biol. 244, 416–423. (doi:10.1016/j.jtbi.2006.08.027) 17064734 10.1016/j.jtbi.2006.08.027

[14] de Lacaze-Duthiers H 1864 Histoire naturelle du corail: organisation, reproduction, pêche en Algérie, industrie et commerce. J.-B. Baillière et fils.

[15] Santangelo G, Carletti E, Maggi E, Bramanti L 2003 Reproduction and population sexual structure of the overexploited Mediterranean red coral *Corallium rubrum*. Mar. Ecol. Prog. Ser. 248, 99–108. (doi:10.3354/meps248099)

[16] Gamble T, Zarkower D 2014 Identification of sex-specific molecular markers using restriction site-associated DNA sequencing. Mol. Ecol. Resour. 14, 902–913. (doi:10.1111/1755-0998.12237) 24506574 10.1111/1755-0998.12237

[17] Gamble T 2016 Using RAD-seq to recognize sex-specific markers and sex chromosome systems. Mol. Ecol. 25, 2114–2116. (doi:10.1111/mec.13648) 27213697 10.1111/mec.13648

[18] Sambrook J, Fritsch EF, Maniatis T, others 1989 Molecular cloning, vol. 2. New York, NY: Cold Spring Harbor Laboratory Press.

[19] Etter PD, Bassham S, Hohenlohe PA, Johnson EA, Cresko WA 2011 SNP discovery and genotyping for evolutionary genetics using RAD sequencing. Mol. Methods Evol. Genet. 772, 157–178. (doi:10.1007/978-1-61779-228-1_9) 10.1007/978-1-61779-228-1_9 PMC3658458 22065437

[20] Catchen J, Hohenlohe PA, Bassham S, Amores A, Cresko WA 2013 Stacks: an analysis tool set for population genomics. Mol. Ecol. 22, 3124–3140. (doi:10.1111/mec.12354) 23701397 10.1111/mec.12354 PMC3936987

[21] Catchen JM, Amores A, Hohenlohe P, Cresko W, Postlethwait JH 2011 *Stacks*: building and genotyping loci *de novo* from short-read sequences. G3 Genes Genomes Genet. 1, 171–182. (doi:10.1534/g3.111.000240) 10.1534/g3.111.000240 PMC3276136 22384329

[22] Jombart T 2008 adegenet: a R package for the multivariate analysis of genetic markers. Bioinformatics 24, 1403–1405. (doi:10.1093/bioinformatics/btn129) 18397895 10.1093/bioinformatics/btn129

[23] R Development Core Team. 2011 R 2.14. 1. R project for statistical computing, Vienna, Austria.

[24] Pratlong M et al., In preparation. Identifying adaptive loci in a context of high genetic structure, local adaptation to depth in the red coral.

[25] Qiu S, Bergero R, Guirao-Rico S, Campos JL, Cezard T, Gharbi K, Charlesworth D 2016 RAD mapping reveals an evolving, polymorphic and fuzzy boundary of a plant pseudoautosomal region. Mol. Ecol. 25, 414–430. (doi:10.1111/mec.13297) 26139514 10.1111/mec.13297

[26] Bergero R, Qiu S, Forrest A, Borthwick H, Charlesworth D 2013 Expansion of the pseudo-autosomal region and ongoing recombination suppression in the *Silene latifolia* sex chromosomes. Genetics 194, 673–686. (doi:10.1534/genetics.113.150755) 23733786 10.1534/genetics.113.150755 PMC3697972

[27] Gautier M, Gharbi K, Cezard T, Foucaud J, Kerdelhué C, Pudlo P, Cornuet J-M, Estoup A 2013 The effect of RAD allele dropout on the estimation of genetic variation within and between populations. Mol. Ecol. 22, 3165–3178. (doi:10.1111/mec.12089) 23110526 10.1111/mec.12089

[28] Kafkas S, Khodaeiaminjan M, Güney M, Kafkas E 2015 Identification of sex-linked SNP markers using RAD sequencing suggests ZW/ZZ sex determination in *Pistacia vera* L. BMC Genomics 16, 98 (doi:10.1186/s12864-015-1326-6) 25765114 10.1186/s12864-015-1326-6 PMC4336685

[29] Carmichael SN et al. 2013 Identification of a sex-linked SNP marker in the salmon louse (*Lepeophtheirus salmonis*) using RAD sequencing. PLoS ONE 8, e77832 (doi:10.1371/journal.pone.0077832) 24147087 10.1371/journal.pone.0077832 PMC3797693

[30] Palaiokostas C et al. 2013 Mapping the sex determination locus in the Atlantic halibut (*Hippoglossus hippoglossus*) using RAD sequencing. BMC Genomics 14, 566 (doi:10.1186/1471-2164-14-566) 23957753 10.1186/1471-2164-14-566 PMC3765698

[31] Palaiokostas C, Bekaert M, Khan MG, Taggart JB, Gharbi K, McAndrew BJ, Penman DJ, Orban L 2013 Mapping and validation of the major sex-determining region in Nile tilapia (*Oreochromis niloticus* L.) using RAD sequencing. PLoS ONE 8, e68389 (doi:10.1371/journal.pone.0068389) 23874606 10.1371/journal.pone.0068389 PMC3708939

[32] Cherif E et al. 2013 Male-specific DNA markers provide genetic evidence of an XY chromosome system, a recombination arrest and allow the tracing of paternal lineages in date palm. New Phytol. 197, 409–415. (doi:10.1111/nph.12069) 23231423 10.1111/nph.12069

[33] Gamble T, Coryell J, Ezaz T, Lynch J, Scantlebury DP, Zarkower D 2015 Restriction site-associated DNA sequencing (RAD-seq) reveals an extraordinary number of transitions among gecko sex-determining systems. Mol. Biol. Evol. 32, 1296–1309. (doi:10.1093/molbev/msv023) 25657328 10.1093/molbev/msv023

[34] Bianchi NO 2002 Akodon sex reversed females: the never ending story. Cytogenet. Genome Res. 96, 60–65. (doi:10.1159/000063029) 12438781 10.1159/000063029

[35] Saunders PA, Franco T, Sottas C, Maurice T, Ganem G, Veyrunes F 2016 Masculinised behaviour of XY females in a mammal with naturally occuring sex reversal. Sci. Rep. 6, 22881 (doi:10.1038/srep22881) 26964761 10.1038/srep22881 PMC4786791

[36] Fowler BL, Buonaccorsi VP 2016 Genomic characterization of sex-identification markers in *Sebastes carnatus* and *S. chrysomelas* rockfishes. Mol. Ecol. 25, 2165–2175. (doi:10.1111/mec.13594) 26923740 10.1111/mec.13594

[37] Chen C-J, Shikina S, Chen W-J, Chung Y-J, Chiu Y-L, Bertrand JAM, Lee Y-H, Chang C-F 2016 A novel female-specific and sexual reproduction-associated Dmrt gene discovered in the stony coral, *Euphyllia ancora*. Biol. Reprod. 94, 40 (doi:10.1095/biolreprod.115.133173) 26740592 10.1095/biolreprod.115.133173

[38] Traylor-Knowles NG, Kane EG, Sombatsaphay V, Finnerty JR, Reitzel AM 2015 Sex-specific and developmental expression of Dmrt genes in the starlet sea anemone, *Nematostella vectensis*. EvoDevo 6, 13 (doi:10.1186/s13227-015-0013-7) 25984291 10.1186/s13227-015-0013-7 PMC4433094

[39] Bergero R, Charlesworth D 2009 The evolution of restricted recombination in sex chromosomes. Trends Ecol. Evol. 24, 94–102. (doi:10.1016/j.tree.2008.09.010) 19100654 10.1016/j.tree.2008.09.010

[40] Pratlong M, Rancurel C, Pontarotti P, Aurelle D In press Monophyly of Anthozoa (Cnidaria): why do nuclear and mitochondrial phylogenies disagree? Zool. Scrip. (doi:10.1111/zsc.12208)

[41] Zapata F et al. 2015 Phylogenomic analyses support traditional relationships within Cnidaria. PLoS ONE 10, e0139068 (doi:10.1371/journal.pone.0139068) 26465609 10.1371/journal.pone.0139068 PMC4605497

[42] Bramanti L et al. 2013 Detrimental effects of ocean acidification on the economically important Mediterranean red coral (*Corallium rubrum*). Glob. Change Biol. 19, 1897–1908. (doi:10.1111/gcb.12171) 10.1111/gcb.12171 23505003

[43] Garrabou J, Perez T, Sartoretto S, Harmelin JG 2001 Mass mortality event in red coral *Corallium rubrum* populations in the Provence region (France, NW Mediterranean). Mar. Ecol. Prog. Ser. 217, 263–272. (doi:10.3354/meps217263)

[44] Priori C, Erra F, Angiolillo M, Santangelo G 2015 Effects of gastropod predation on the reproductive output of an overexploited deep octocoral. Coral Reefs 34, 59–63. (doi:10.1007/s00338-014-1223-5)

[45] Pratlong M (2016). Data from: Evidence for a genetic sex determination in Cnidaria, the Mediterranean red coral (*Corallium rubrum*). Short Read Archive (SRA) database. Accessions no. SRR5186771-SRR5187129..

[46] Pratlong M et al. 2017 Data from: Evidence for a genetic sex determination in Cnidaria, the Mediterranean red coral (*Corallium rubrum*). Dryad Digital Repository (doi:10.5061/dryad.rs7bm) 10.1098/rsos.160880 PMC5383831 28405374

