# Supplementary material for: Evidence for a genetic sex determination in Cnidaria, the Mediterranean red coral (Corallium rubrum)
Source: R Soc Open Sci. 2017 Mar 1;4(3):160880. doi: 10.1098/rsos.160880 (PMC5383831; doi:10.1098/rsos.160880)
Supplement: Supplementary figures [file rsos160880supp1.pdf]

SUPPLEMENTARY FIGURES

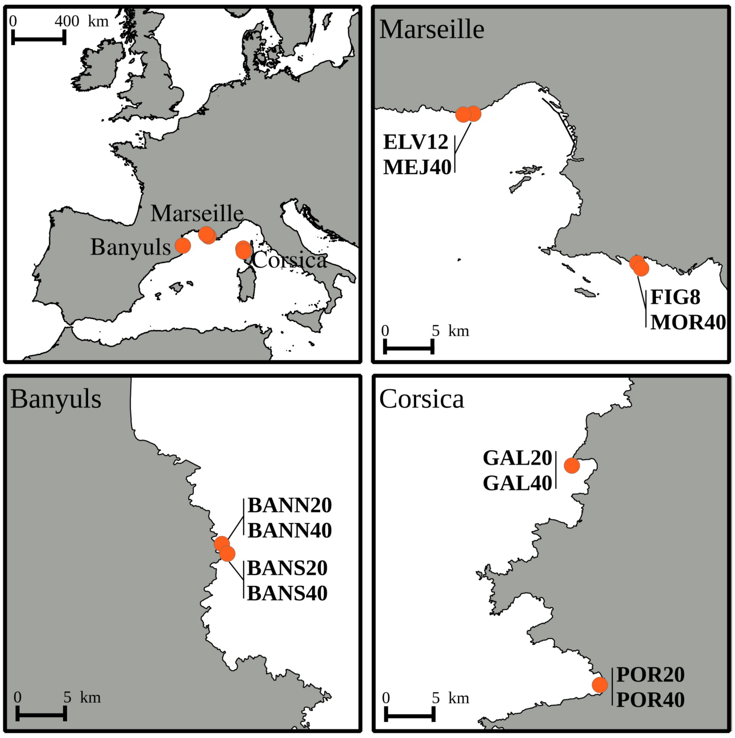

Fig. S1. Locations of the sampling sites of the red coral individuals among the studied three geographical regions.

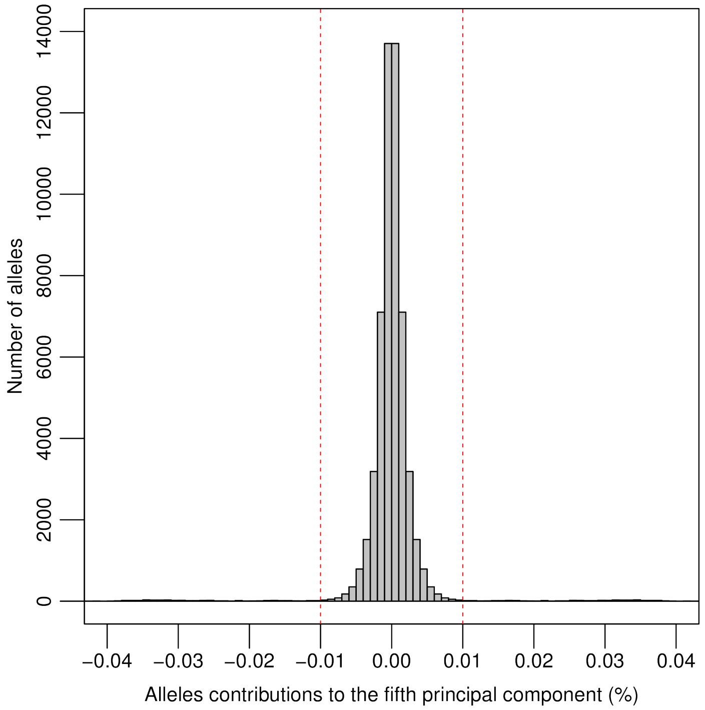

Fig. S2. Loci contribution to the fifth axis of the PCA (see Fig. 2). The dash line indicate the threshold of selected loci.

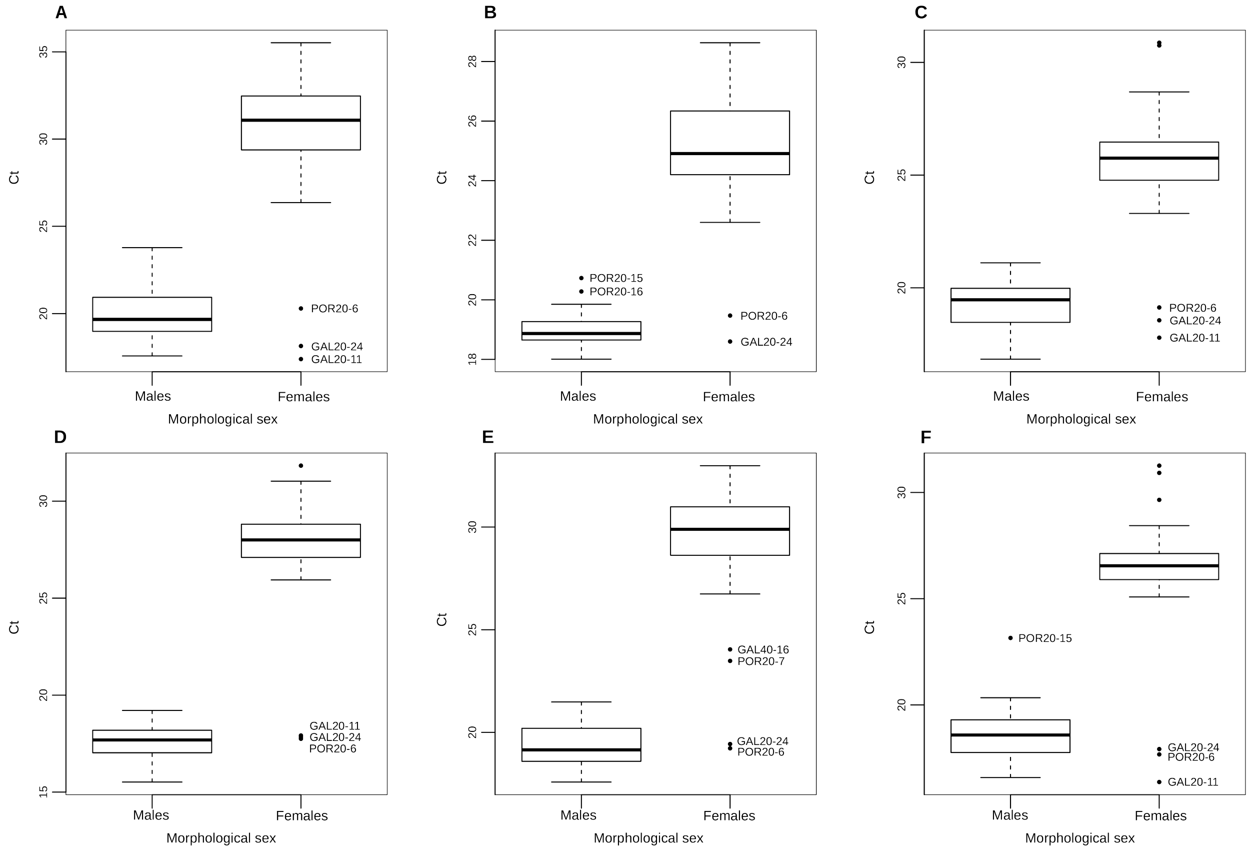

Fig. S3. Result of the real-time PCR (threshold cycle, Ct) of six male-specific loci on morphologically sexed males and females. The Ct indicating the number of PCR cycles necessary to reach a threshold value, a low Ct value indicates a high amplification rate from the corresponding sample. **A**. Locus_139082. **B**. Locus_144530. **C**. Locus_147437. **D**. Locus_139282. **E**. Locus_158229. **F** Locus_154834.

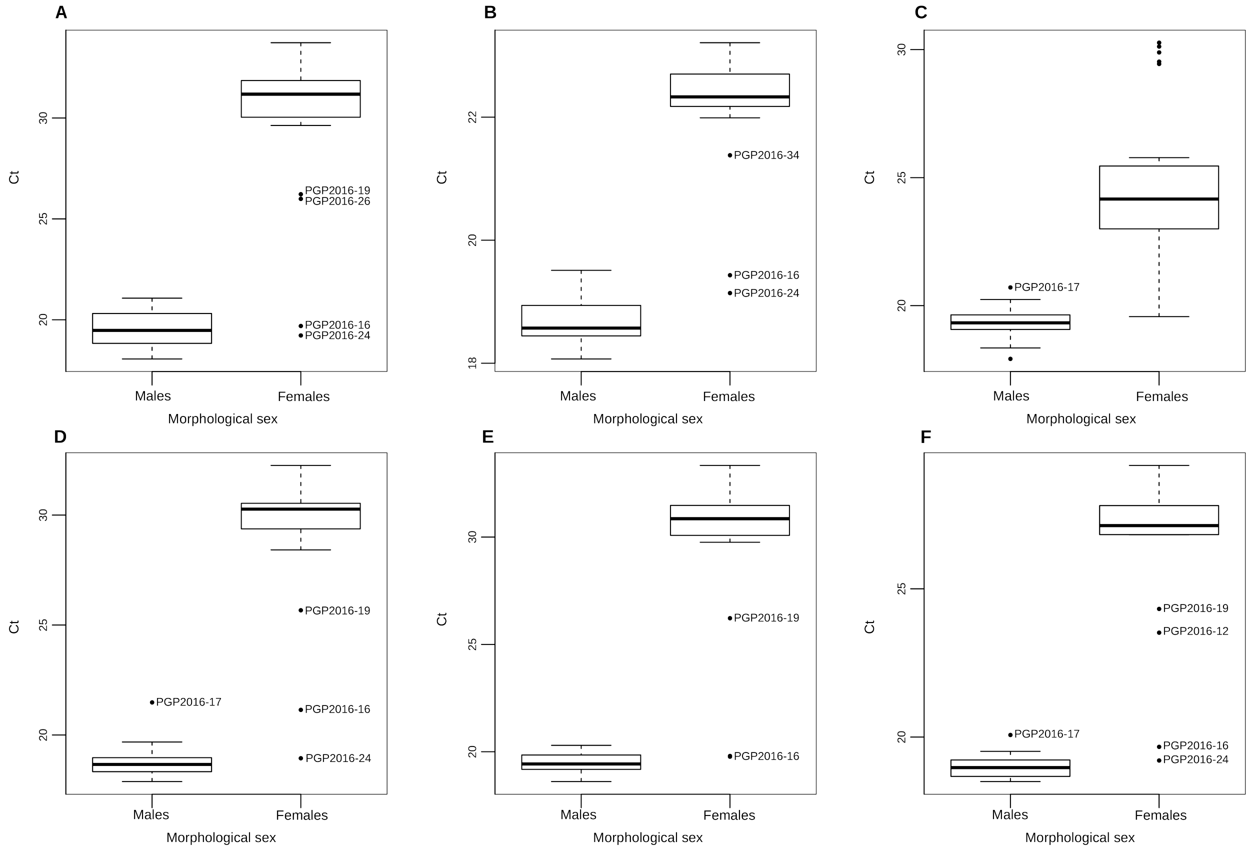

Fig. S4. Result of the real-time PCR (threshold cycle, Ct) of six male-specific loci on morphologically sexed males and females from the independent validation. The Ct indicating the number of PCR cycles necessary to reach a threshold value, a low Ct value indicates a high amplification rate from the corresponding sample. **A**. Locus_139082. **B**. Locus_144530. **C**. Locus_147437. **D**. Locus_139282. **E**. Locus_158229. **F**. Locus_154834.
