# Supplementary material for: Evidence for a genetic sex determination in Cnidaria, the Mediterranean red coral (Corallium rubrum)
Source: R Soc Open Sci. 2017 Mar 1;4(3):160880. doi: 10.1098/rsos.160880 (PMC5383831; doi:10.1098/rsos.160880)
Supplement: Supplementary tables [file rsos160880supp2.pdf]

SUPPLEMENTARY TABLES

Table S1. Characteristics of red coral sampling sites.

Population	Geographic region	Depth (m)	GPS	GPS
FIG8	Marseille	8	43° 12.330'N	5° 26.790'E
MOR40	Marseille	40	43° 12.060'N	5° 27.100'E
ELV12	Marseille	12	43° 19.780'N	5° 14.210'E
MEJ40	Marseille	40	43° 19.700'N	5° 13.480'E
BANN20	Banyuls	25	42° 26.890'N	3° 10.330'E
BANN40	Banyuls	35	42° 26.890'N	3° 10.330'E
BANS20	Banyuls	26	42° 26.390'N	3° 10.790'E
BANS40	Banyuls	36	42° 26.390'N	3° 10.790'E
POR20	Corsica	21	42° 16.292'N	8° 41.255'E
POR40	Corsica	33	42° 16.292'N	8° 41.255'E
GAL20	Corsica	26	42° 28.210'N	8° 38.950'E
GAL40	Corsica	36	42° 28.210'N	8° 38.950'E
PGP2016	Marseille	10	43° 11.190'N	5° 23.470'E

Table S2. Counts of SNP loci after each step of filtering.

Step	Number of SNPs	Software
After assembly raw data	138 810	Stacks (1,2)
Excluding loci not in within population HWE	86 520	VCFtools (3)
MAF 1 %	56 844	VCFtools (3)
One SNPs per RAD-tag	27 461	

Table S3. Primers sequences of male-specific markers tested by real-time PCR.

Marker name	Primers sequences
Locus_139082	F TTCCAGGTATATGCCTTTCCATAA<br>R GTGAGCCCAAATCCCTCAA
Locus_147437	F AAGATCAACTCGACACCACG<br>R TGTACAGGCCAACAATAATTCCA
Locus_144530	F GCAGAGCCGTTCACCTTGAA<br>R CCTCTCCGACTAACCATCC
Locus_139282	F GATTCTCCTGCTAGAGCAATTGTA<br>R CCCTTCAAACTCACACCAGTC
Locus_158229	F CTASTTTGTGCAATAAATGTGCCT<br>R GTACAAGTCCTAGAGCTCAGAT
Locus_154834	F TGCAGATGCCATGCCTTAAAAAAT<br>R TCCGTTTTCGTTTTTCAGGTGCAT

Table S4. Results of the process_radtags program of the Stacks pipeline (2,3).

Library	Retained reads (%)	Low quality (%)	Ambiguous barcodes (%)	Ambiguous RAD-Tag (%)	Number of total rad-tags
1	96.60	0.13	1.35	1.92	211 852 559
2	96.99	0.11	1.42	1.48	188 028 997
3	90.93	4.71	2.66	1.69	183 805 971
4	97.13	0.11	1.41	1.34	211 503 256
5	95.49	0.61	1.36	2.55	204 047 518
6	96.20	0.60	1.37	1.83	202 415 269
7	85.09	9.66	3.30	1.96	179 654 122
8	96.23	0.63	1.30	1.85	207 675 337
9	94.31	1.54	1.44	2.71	215 949 328
10	95.15	0.41	1.81	2.63	154 386 898
11	95.71	0.40	1.59	2.31	158 810 476
12	96.23	0.42	1.32	2.03	195 832 367

Table S5. Sex-ratio of red coral populations considering the PCA-identified males and females (see Fig. 2). Differences from balanced sex-ratio were tested by a chi2 test.

Population	Number of females	Number of males	p
BANN20	11	19	0.14
BANN40	15	13	0.71
BANS20	13	17	0.47
BANS40	17	13	0.47
ELV12	9	21	0.03
MEJ40	17	12	0.35
FIG8	17	13	0.47
MOR40	13	17	0.47
GAL20	12	16	0.45
GAL40	15	14	0.85
POR20	14	16	0.72
POR40	16	14	0.72
Total	169	185	0.40

Table S6. p-values of the Wilcoxon–Mann–Whitney test of amplification differences between males and females (real-time PCR experiment).

Locus	P-values, Corsica individuals	P-values, Marseille individuals
Locus_139082	$3.48.10^{-8}$	$4.81.10^{-6}$
Locus_144530	$8.87.10^{-9}$	$6.11.10^{-7}$
Locus_147437	$1.98.10^{-7}$	$6.85.10^{-7}$
Locus_139282	$2.86.10^{-8}$	$6.11.10^{-7}$
Locus_158229	$2.41.10^{-11}$	$1.11.10^{-8}$
Locus_154834	$2.96.10^{-8}$	$5.46.10^{-7}$

SUPPLEMENTAL REFERENCES

1. Catchen JM, Amores A, Hohenlohe P, Cresko W, Postlethwait JH. Stacks: building and genotyping loci de novo from short-read sequences. G3 Genes Genomes Genet. 2011;1(3):171–182.
2. Catchen J, Hohenlohe PA, Bassham S, Amores A, Cresko WA. Stacks: an analysis tool set for population genomics. Mol Ecol. 2013;22(11):3124–3140.
3. Danecek P, Auton A, Abecasis G, Albers CA, Banks E, DePristo MA, et al. The variant call format and VCFtools. Bioinformatics. 2011;27(15):2156-8.
