# Supplementary material for: Evidence for a genetic sex determination in Cnidaria, the Mediterranean red coral (Corallium rubrum)
Source: R Soc Open Sci. 2017 Mar 1;4(3):160880. doi: 10.1098/rsos.160880 (PMC5383831; doi:10.1098/rsos.160880)
Supplement: Supplementary methods (libraries preparation and analysis of RAD-Sequencing and real-time PCR protocol) [file rsos160880supp3.pdf]

SUPPLEMENTARY METHODS

RAD-Sequencing

Twelve RAD libraries were prepared according to the protocol described in (1), with small modifications. Briefly, 1 µg of genomic DNA for each sample was digested using high-fidelity PstI during 60 min at 37 °C. P1 adapters, with 4-6 bp individual barcodes were then ligated to each sample using 0.5 µL of T4 DNA ligase (NEB), 0.5 µL of rATP 100 mM (Promega), 1 µL of DTT 500 mM (Promega), 1 µL of 10X T4 ligase buffer (NEB) and incubated during 60 min at 22 °C, 10 min at 65 °C and 1 min at 64 °C. DNA samples were pooled by 32 (generally by location), sheared, size selected and P2-barcoded. Final PCR for RAD-tags enrichment were performed with 16 cycles and primers dimers were removed during a final AMPure Beads Purification (Agencourt). Libraries were sequenced on an Illumina HiSeq2000 using 100 bp single-end reads, at the Biology Institute of Lille (IBL, UMR 8199 CNRS) and at the MGX sequencing platform in Montpellier (France).

The STACKS pipeline (2,3) was used for the loci *de novo* assembly and genotyping. Quality filtering and demultiplexing were performed with the *process_radtags* module with default parameters which enables us in particular to remove any read with uncalled base and to performe a phred-33 quality filtering of raw reads. Exact-matching RAD loci (putative orthologous tags) were individually assembled using *ustacks* with a minimum depth of coverage of five reads per allele (m = 5) and a maximum of five nucleotide mismatches between allele (M = 5). These parameters were optimized during preliminary runs. *Cstacks* was used to build a catalogue of consensus loci from all individuals, with five mismatches allowed between individuals at the same locus (n = 5). Matches of individual RAD loci to the catalogue of loci were searched using *sstacks*. Finally, the *population* module was used to obtain the loci that were successfully genotyped in at least 75 % of individuals from all populations. Because of the increased of error rates toward the end of the reads,

we observed an increase in the number of SNPs from position 86 bp to 91 bp and we removed these positions from the analysis. In order to filter for poor-quality SNPs and artifacts due paralogous sequences, we used VCFtools (4) to remove SNPs that were not at the Hardy-Weinberg equilibrium within at least one of the 12 populations with a p-value threshold of 0.01 (Table S2). SNPs with a minor allele frequencies below 0.01 were removed using VCFTools (Table S2). Individuals with more than 30 % of missing genotypes were discarded. Finally, only the first SNP of each RAD-tag was kept for further analysis (Table S2).

Finally, and in order to identify potential locus presenting known function of sex determination, we mapped the loci of the Stacks catalogue against the *Corallium rubrum* transcriptome (5) using BWA (version 0.7.12; (6)) and Samtools (version 1.3; (7)) with default parameters. Functional annotation of these loci was obtained using BLAST 2GO (8).

Real-time PCR

Real-time PCRs were performed on Eppendorf Master cycler, using Invitrogen platinium SYBRGreen QPCR supermix (final volume 25 µL, primers concentration 400 nM). PCR efficiency was checked with dilution range from 1 to 1 000, and specificity with melting curve analysis. PCR program consisted of initial enzyme activation (50 °C 2 min), denaturation (95 °C 2 min), 40 cycles (95 °C 15 s / 52 °C 30 s / 60 °C 30 s) and melting curve protocole. Ct values were measured for the same normalized fluorescence threshold between all experiments.

SUPPLEMENTAL REFERENCES

1. Etter PD, Bassham S, Hohenlohe PA, Johnson EA, Cresko WA. SNP discovery and genotyping for evolutionary genetics using RAD sequencing. Mol Methods Evol Genet. 2011;157–178.
2. Catchen JM, Amores A, Hohenlohe P, Cresko W, Postlethwait JH. Stacks: building and genotyping loci de novo from short-read sequences. G3 Genes Genomes Genet. 2011;1(3):171–182.
3. Catchen J, Hohenlohe PA, Bassham S, Amores A, Cresko WA. Stacks: an analysis tool set for

population genomics. Mol Ecol. 2013;22(11):3124–3140.

4. Danecek P, Auton A, Abecasis G, Albers CA, Banks E, DePristo MA, et al. The variant call format and VCFtools. Bioinformatics. 2011;27(15):2156-8.
5. Pratlong M, Haguenauer A, Chabrol O, Klopp C, Pontarotti P, Aurelle D. The red coral (*Corallium rubrum*) transcriptome: a new resource for population genetics and local adaptation studies. Molecular ecology resources. 2015;15(5):1205-1215.
6. Li H, & Durbin R. Fast and accurate short read alignment with Burrows–Wheeler transform. Bioinformatics. 2009;25(14):1754-1760.
7. Li H, Handsaker B, Wysoker A, Fennell T, Ruan J, Homer N, et al. The sequence alignment/map format and SAMtools. Bioinformatics. 2009;25(16):2078-2079.
8. Conesa A, Götz S, García-Gómez JM, Terol J, Talón M, Robles M. Blast2GO: a universal tool for annotation, visualization and analysis in functional genomics research. Bioinformatics. 2005;21(18):3674-3676.
